# Longitudinal Relationships between Posttraumatic Stress Symptoms and Sleep Problems in Adolescent Survivors following the Wenchuan Earthquake in China

**DOI:** 10.1371/journal.pone.0104470

**Published:** 2014-08-08

**Authors:** Xiao Zhou, Xinchun Wu, Yuanyuan An, Fang Fu

**Affiliations:** 1 School of Psychology, Beijing Normal University, Beijing, People's Republic of China; 2 School of Psychology, Nanjing Normal University, Nanjing, People's Republic of China; 3 School of Social Development and Public Policy, Fudan University, Shanghai, People's Republic of China; Univ of Toledo, United States of America

## Abstract

**Purpose:**

To examine the longitudinal relationships between Posttraumatic Stress Disorder (PTSD) and sleep problems among adolescent survivors in the Wenchuan earthquake, China.

**Methods:**

350 adolescent survivors were randomly selected from several primary and secondary schools in the counties of Wenchuan and Maoxian, the two areas most severely affected by the Wenchuan earthquake. Participants completed Revised Child PTSD Symptom Scale and Sleep Problems Subscale of Self-generated Child Behavior Problems Questionnaire at one year (T1), one-and-a-half years (T2), two years (T3) after the earthquake, respectively.

**Results:**

There was a bidirectional relationship between intrusive symptom clusters of PTSD and sleep problems from T1 to T2, and this relationship became non-significant from T2 to T3. There was a one-way predictive relationship of avoidance symptom clusters of PTSD onto sleep problems from T1 to T3. The hyperarousal symptom clusters of PTSD had effects on sleep problems from T1 to T2 but not from T2 to T3, while sleep problems have no significant effect on hyperarousal symptom clusters of PTSD from T1 to T3. In addition, the relationships between three symptom clusters of PTSD and sleep problems weakened with time change.

**Conclusions:**

From 1 year to 1.5 years after the earthquake, all the three symptom clusters of PTSD could be important predictive factors for the development and maintenance of sleep problems, while sleep problems could only be risk factors for the intrusive symptom clusters of PTSD. From 1.5 years to 2 years, only the avoidance symptom clusters of PTSD were risk factors for sleep problems, and sleep problems had no significant effects on any symptom clusters of PTSD. Overall, the relationship between PTSD and sleep problems weakened with time change.

## Introduction

As one of the most destructive of natural disasters, the earthquake that occurred in Wenchuan, China in 2008 caused widespread life and property losses. Adolescent survivors of this earthquake experienced significant psychological reactions; posttraumatic stress disorder (PTSD) is often considered to be the most frequent psychological reactions in the aftermath of disasters [1]. More importantly, adolescents' PTSD symptoms were found to be related to sleep problems (e.g., difficulty falling asleep, sleepless nights and early-morning wakefulness) after disaster [2]. Some researchers have even suggested that PTSD commonly co-occur with sleep problems [3], and indicated that there is a consistent relation between PTSD and sleep problems after trauma [4], [5]. Among those with PTSD, symptom severity positively co-varies with self-reported sleep problems [6]. For example, among a sample of 2853 adolescent survivors after disaster, increases in sleep problems paralleled increases in PTSD symptom severity [4]. These studies mainly examined the relationship between global PTSD levels and severity of sleep problems among individuals with PTSD. The global PTSD, in the DSM-IV-TR, includes three specific types of symptoms: re-experiencing (intrusive) symptoms (e.g., nightmares, flashbacks), avoidance symptoms (e.g., avoiding reminders of the event), and hyperarousal symptoms (e.g., increased startle response, anger outbursts) [7].

Nevertheless, although the PTSD/sleep problems co-occurrance is prevalent in the aftermath of trauma, the specific nature of this association remains unclear. To some extent, Mellman suggested that it could be attributed to the overlap between sleep problems and PTSD [8]. For example, sleep problems such as insomnia and nightmare are criteria to diagnose PTSD and even the core features of PTSD [9]. However, some studies which excluded some items of sleep problems in the symptom clusters of PTSD and controlled the overlapped parts also found that PTSD co-occurred with sleep problems [10], [11]. These results have indicated that the PTSD/sleep problems co-occurrance is not merely attributed to their overlap. Thus, multiple theories have been proposed to account for the global association between PTSD and sleep problems [12].

The emotional processing theory of traumatic stress exposure posits that PTSD patients' traumatic memory has a particular fear structure that is thought to be stable and broadly generalized so that it is easily accessed, even during sleep [13]. Once this fear structure is activated by re-experiencing traumatic related clues during sleep, it would exacerbate PTSD patients' fear about sleep, which in turn makes them avoid going to sleep and results in sleep problems. From the perspective of the theory, it is more likely that intrusive symptom clusters of PTSD are related to sleep problems. Other theoretical work aiming to explain the PTSD-sleep problems co-occurrance is hyperarousal-based theories [14], which suggest that PTSD patients' sleep may be disrupted by hyperarousal states, which can increase the level of awareness and result in sensitive responses to the external environment, and in turn break the necessary condition of sleep [15] and cause PTSD patients' sleep difficulties or insomnia. Based on hyperarousal theories in this domain, it is possible that hyperarousal symptom clusters of PTSD would evidence relations with sleep problems.

Relatively limited theoretical and empirical work has suggested a model for the role of avoidance symptom clusters of PTSD in sleep problems [3]. While some researchers have supposed that people with elevated levels of PTSD endorse relatively elevated use of control-oriented strategies, including avoidance, to manage cognitive-affective experiences [16]. These attempts are counter productive and actually increase cognitive and physiological arousal that impede sleep onset [17], thereby result in sleep problems. Thus, it is possible that avoidance symptom clusters of PTSD are related greater to sleep problems.

In contrast, a further hypothesis suggests that PTSD symptoms themselves are a consequence of sleep problems rather than a causal factor. The view has recently been supported by many empirical studies [2], [18], [19]. Two possible mechanisms have been offered to explain the role of sleep problems in global PTSD. First potential explanation is a leading theoretical model provided by Rothbaum and Mellman [20], the model suggests that the daytime consequences of sleep problems (elevations in daytime fatigue, confusion, tension and anxiety) can generate a context in which trauma survivors are more sensitive and reactive to trauma reminders. For this reason, individuals are more likely to avoid these reminders (thereby maintaining posttraumatic stress symptoms) and react with greater anxiety to trauma reminders when they cannot be avoided [21]. The second hypothesis supposes that sleep problems can reduce sleep quality and/or quantity, result in poor concentration, agitation/irritability, and impair emotional coping, which may lead to more frequent and intense negative emotions (e.g., anger, sadness, and anxiety) in the short term and more frequent emotional complaints (depression and anxiety) in the long term [22]. For this reason, individuals with sleep problems who have experienced an extremely critical event (e.g., traumatic event) may have more difficulties in processing the event and may be more likely to suffer from PTSD [9]. From the two potential explanations, it is likely that sleep problems also may be related to all the three symptom clusters of PTSD.

Although several theoretical predictions have been advanced to describe the relationship between specific PTSD symptom clusters and sleep problems, the predictive utility of these theories focused on various aspects of the PTSD has not been evaluated. Furthermore, prior studies do not allow for conclusions regarding the causal relationships between specific PTSD symptom clusters and sleep problems because of cross-sectional design [12]. Many researchers have emphasized that it is necessary to carry out a longitudinal study on the relationship between PTSD symptom clusters and sleep problems, and to determine causal relationships between PTSD symptom clusters and sleep problems [3], [23]. Moreover, previous studies have suggested that the relationship between PTSD and sleep problems can change with time [24], but fewer studies have been done to examine it among adolescents after disaster. The importance of extending these literatures to youth is evidenced by the high rates of traumatic event exposure [25] and sleep problems [26] within this developmental period.

Given the backdrop, the study aimed to examine whether the three symptom clusters of PTSD can predict sleep problems; to assess whether sleep problems can predict the three symptom clusters of PTSD; to determine whether the three symptom clusters of PTSD and sleep problems can predict each other over time. To address these aims, we tested two specific hypotheses and an exploratory hypothesis. First, based on previous theories [13], [14], [16], we tested the hypothesis that all the three symptom clusters of PTSD could predict sleep problems. Second, according to Rothbaum and Mellman' theoretical model [20], it was predicted that sleep problems could affect three symptom clusters of PTSD. Finally, exploratory analyses were conducted to examine whether the relationships between three symptom clusters of PTSD and sleep problems would change with time or not. To test these hypotheses, we built three cross-lagged models between intrusive symptoms and sleep problems, avoidance symptoms and sleep problems, and hyperarousal symptoms and sleep problems respectively.

## Method

### Participants and Procedures

In the present study, 350 adolescent survivors were randomly selected from several primary and secondary schools in the counties of Wenchuan and Maoxian, the two areas most severely affected by the Wenchuan earthquake. All adolescents have no psychiatric conditions prior to the earthquake. The mean age of the adolescents was 15.65 (SD = 1.74) years at the first measurement wave, with ages ranging from 12.0 to 19.0 years. Of the 350 participants, 201 (57.4%) were female and 149 (42.6%) were male. With respect to ethnicity, 67 (19.1%) belonged to the Han ethnic group, 87 (24.9%) belonged to the Tibetan ethnic group, 188 (53.7%) belonged to the local Qiang group, and 8 (2.3%) belonged to other minor ethnic categories.

This project was approved by the Research Ethics Committee of Beijing Normal University and the local education authorities (i.e., County Departments of Education) as well as the participating school principals. Written informed consent was obtained from school principals and classroom teachers. In China, research projects that are approved by local education authorities and the school administrators and that are deemed to provide a service to the students do not require parental consent. Thus, the current project was not required to obtain written informed consent from parents. The purpose of the study and the autonomy of the students were highlighted before the survey. Written informed consent was obtained from each subject, and the right to withdraw from the survey at any time was provided for them. Three assessments were conducted at different time points under the supervision of trained individuals with Master's degree in psychology. No compensations were provided to the students for their participation other than possible counseling if needed.

Of 350 participants, all the participants completed the first assessment at one year after the earthquake (T1). At the second assessment, a year and a half after the earthquake (T2), 334 (95.4%) of the original 350 sample completed the survey; 279 (77.4%) completed the third assessment at two years after the earthquake (T3). During the course of the follow-up, all adolescents were free of medications as well as drug abuse. In each follow-up survey, some students dropped out the school or graduated from the school, thus there are some drop-out rates. To investigate the potential impact of attrition, we tested the differences in demographic variables (e.g., age, gender and ethnicity) and the main study variables (i.e., three symptom clusters of PTSD and sleep problems) from the first assessment between the longitudinal sample and the subjects who did not follow up for unknown reasons (i.e., other than dropout and graduation). Attrition analysis results showed that except for age [χ^2^(7) = 15.38, p<0.05], there were no significant differences in gender [χ^2^(1) = 1.03, p>0.05], ethnicity [χ^2^(3) = 4.40, p>0.05], intrusive symptom clusters of PTSD [*t*(348) = 0.86, p>0.05], avoidance symptom clusters of PTSD [*t*(348) = 0.60, p>0.05], hyperarousal symptom clusters of PTSD [*t*(348) = 0.78, p>0.05] and sleep problems [*t*(348) = 0.63, p>0.05].

### Measures

#### The Child PTSD Symptom Scale

Posttraumatic stress symptoms were assessed with the Child PTSD Symptom Scale [27], a 17-item self-reported scale designed to measure the occurrence and frequency of PTSD symptoms according to the Diagnostic and Statistical Manual of Mental Disorders in relation to the most distressing event. In the current study, all the items were translated into Chinese, children rated the frequency of symptoms during the previous two weeks on a 4-point-Likert scale of 0 (not at all/only at one time) to 3 (almost always 5 or more times a week). Subscale scores ranged from 0 to 15 for intrusion, 0 to 21 for avoidance, and 0 to 15 for hyperarousal. The overall severity score was generated by adding the scores of all three subscales. In the current sample, the scale exhibited good internal consistency (alpha coefficient for global PTSD was 0.89 at T1, 0.90 at T2 and 0.91 at T3; the alpha coefficients for intrusive symptoms, avoidance symptoms and hyperarousal symptoms were 0.80, 0.77 and 0.80 at T1; 0.71, 0.74 and 0.78 at T2; 0.75, 0.79 and 0.83 at T3, respectively) and good fit indices in confirmative factor analysis (χ^2^/df = 2.21, NFI = 0.87, CFI = 0.93, RMSEA = 0.059).

#### The Child Behavior Problems Questionnaire

The Child Behavior Problems Questionnaire was generated by (a) evaluating the adolescents' circumstances after the Wenchuan earthquake and (b) revising the Youth Risk Behavior Survey Questionnaire [28]. This questionnaire included a seven factor structure with 19 items assessing behavior problems. The seven factor structure included conflict behaviors, sleep problems, suicidal ideation, dietary behaviors, drug addiction, networked behaviors and related negative behaviors. The response scale ranged from 0 (I did not experience this change) to 2 (I experienced this change a great deal). In the current study, we used the sleep problems subscale, which had 3 items, including difficulty in sleep, sleepless nights and early-morning wakefulness. The subscale demonstrated good internal consistency (alpha coefficient was 0.74 at T1, 0.72 at T2 and 0.94 at T3).

## Data Analysis

Descriptive analyses were conducted to measure levels of intrusive, avoidance, and hyperarousal symptom clusters and sleep problems. Pearson correlations were calculated to examine the associations among intrusive, avoidance, and hyperarousal symptom clusters with sleep problems, both cross-sectionally and longitudinally.

Statistical analyses were conducted using AMOS 7.0 software [29]. Missing data were handled with maximum likelihood estimation with robust standard errors (MLR). To evaluate model fit, we used chi-squared values, the comparative fit index (CFI), Tucker-Lewis index (TLI), normed fit index (NFI) and the root mean square error of approximation (RMSEA). A non-significant chi-squared value indicates a good fit between the model and the data. The general cutoffs for accepting a model were a NFI, CFI and a TLI equal to or greater than 0.90, an RMSEA of equal to or less than 0.08 [30].

To assess the bidirectional relationships among intrusive, avoidance, and hyperarousal symptom clusters of PTSD and sleep problems, the SEM approach was implemented in a cross-lagged model. Regarding that the probability of making a type I error may be exaggerated by the relatively high correlations between the three symptom clusters of PTSD, we controlled the other two symptom clusters of PTSD when analyzing the relationship between one symptom clusters of PTSD and sleep problems.

## Results

### Descriptive Statistics and Correlations

The descriptive statistics and correlations among the various measures are shown in Table 1. The mean levels of intrusive, avoidance and hyperarousal symptoms at Time 1, 2 and 3 were 1.08, 0.76 and 0.80; 0.85, 0.78 and 0.89; 1.01, 0.97 and 1.16, respectively. The mean levels of sleep problems at Time 1, 2 and 3 were 1.76, 1.58 and 1.78, respectively. Next, *pearson's* correlations were calculated between the three PTSD symptom clusters and sleep problems both cross-sectionally and longitudinally. The analyses yielded significant relationships between sleep problems and the three PTSD symptoms at T1, T2 and T3.

**Table 1:** Means, standard deviation and correlations among intrusive, avoidance, hyperarousal symptoms and sleep problems.

		1	2	3	4	5	6	7	8	9	10	11	M	SD
1	T1 Intrusion	1											1.08	0.65
2	T2 Intrusion	0.59**	1.00										0.76	0.55
3	T3 Intrusion	0.27**	0.32**	1.00									0.80	0.74
4	T1 Avoidance	0.80**	0.60**	0.33**	1.00								0.85	0.61
5	T2 Avoidance	0.62**	0.66**	0.23**	0.53**	1.00							0.78	0.51
6	T3 Avoidance	0.33**	0.30**	0.79**	0.33**	0.33**	1.00						0.89	0.69
7	T1 Hyperarousal	0.72**	0.46**	0.23**	0.69**	0.55**	0.28**	1.00					1.01	0.67
8	T2 Hyperarousal	0.61**	0.71**	0.29**	0.58**	0.69**	0.28**	0.59**	1.00				0.97	0.58
9	T3 Hyperarousal	0.27**	0.22**	0.78**	0.29**	0.25**	0.80**	0.30**	0.29**	1.00			1.16	0.73
10	T1 Sleep Problems	0.49**	0.42**	0.33**	0.57**	0.37**	0.35**	0.50**	0.44**	0.33**	1.00		1.76	1.53
11	T2 Sleep Problems	0.45**	0.46**	0.27**	0.36**	0.49**	0.28**	0.42**	0.50**	0.24**	0.48**	1.00	1.58	1.48
12	T3 Sleep Problems	0.30**	0.25**	0.34**	0.31**	0.27**	0.32**	0.27**	0.26**	0.40**	0.40**	0.31**	1.78	1.51

**p<0.01.

### The Relationships among Intrusive Symptoms, Avoidance Symptoms, Hyperarousal Symptoms and Sleep Problems

**Figure 1:**
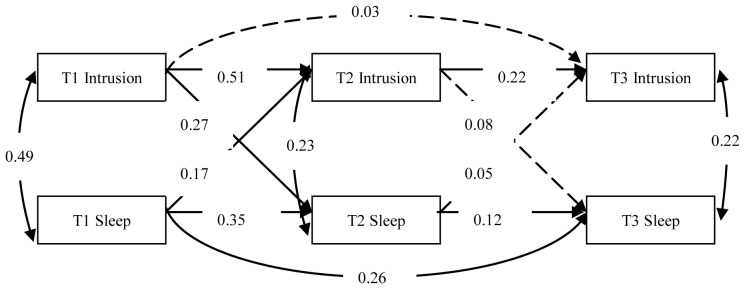
The model for the evaluation of the relationship between intrusive symptoms and sleep problems after controlling the other two symptom clusters of PTSD (Model 1). Dashed lines indicate insignificant paths. Intrusive symptoms = Intrusion, Sleep problems = Sleep.

To examine the bidirectional relationships between specific symptom clusters of PTSD and sleep problems, we established three structural cross-lagged models. And in all the three models, the other two symptom clusters of PTSD were controlled when analyzing the relationship between one symptom clusters of PTSD and sleep problems. Then, the bidirectional effects between intrusive symptoms and sleep problems, between avoidance symptoms and sleep problems, and between hyperarousal symptoms and sleep problems were evaluated by Model 1, Model 2 and Model 3, respectively. In the three models, all structural coefficients were freely estimated and the model fit was good (Figure 1 to Figure 3).

**Figure 2:**
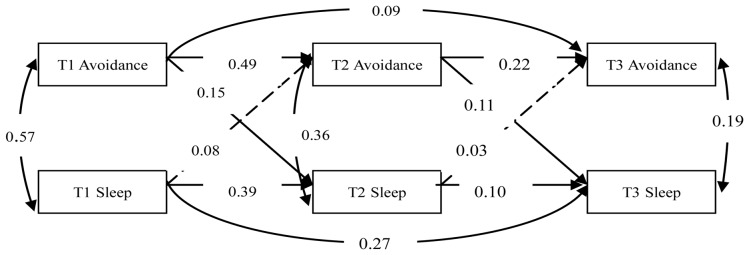
The model for the evaluation of the relationship between avoidance symptoms and sleep problems after controlling the other two symptom clusters of PTSD (Model 2). Dashed lines indicate insignificant paths. Avoidance symptoms = Avoidance, Sleep problems = Sleep.

**Figure 3:**
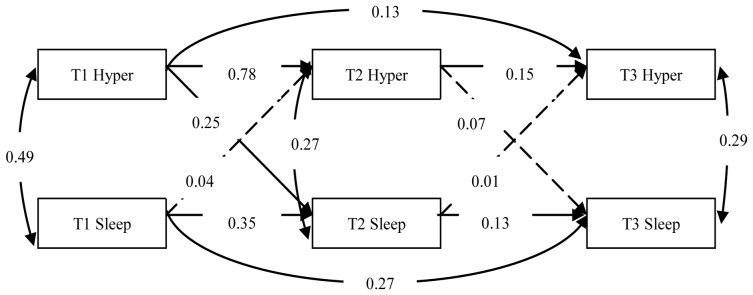
The model for the evaluation of the relationship between hyperarousal symptoms and sleep problems after controlling the other two symptom clusters of PTSD (Model 3). Dashed lines indicate insignificant paths. Hyperarousal symptoms = Hyper, Sleep problems = Sleep.

In Model 1, the model fit was good (χ^2^/df = 2.615, NFI = 0.972, CFI = 0.983, TLI = 0.960, RMSEA = 0.068). Path analyses revealed that intrusive symptoms and sleep problems were associated with each other at T1, T2 and T3. Cross-lagged paths representing the influence of intrusive symptoms on sleep problems indicated that intrusive symptoms at T1 significantly predicted sleep problems at T2 (*γ = 0.27, p<0.01*). Sleep problems at T1 also significantly predicted intrusive symptoms at T2 (*γ = 0.17, p<0.01*). Intrusive symptoms at T2 could not predict sleep problems at T3 (*γ = 0.08, p>0.05*), and neither could sleep problems at T2 predict intrusive symptoms at T3 (*γ = 0.05, p>0.05*).

Considering the content overlap between sleep problems and intrusive symptoms (nightmares), we conducted a structural model analysis again after omitting one intrusive symptom item that was conceptually related to sleep problems. The model fit was virtually unaltered (χ^2^/df = 2.681, NFI = 0.975, CFI = 0.984, TLI = 0.962, RMSEA = 0.069). The stability coefficients were 0.60, 0.19 and 0.07 for intrusive symptoms, and 0.38, 0.12 and 0.27 for sleep problems. The cross-lagged coefficients were 0.16 and 0.07 for the effect of intrusive symptoms on sleep problems, and 0.11 and 0.01 for the effect of sleep problems on intrusive symptoms.

In Model 2, the model fit was good (χ^2^/df = 3.157, NFI = 0.969, CFI = 0.978, TLI = 0.947, RMSEA = 0.079). Path analyses revealed that avoidance symptoms and sleep problems were associated with each another at T1, T2 and T3. Cross-lagged paths representing the influence of avoidance symptoms on sleep problems showed that avoidance symptoms at T1 and T2 significantly predicted sleep problems at T2 (*γ = 0.15, p<0.05*) and T3 (*γ = 0.11, p<0.05*). Sleep problems at T1 and T2 had no significant effect on avoidance symptoms at T2 (*γ = 0.08, p>0.05*) and T3 (*γ = 0.03, p>0.05*).

In Model 3, the model fit was good (χ^2^/df = 2.992, NFI = 0.968, CFI = 0.978, TLI = 0.951, RMSEA = 0.076). Path analyses revealed that hyperarousal symptoms and sleep problems were associated with each other at T1, T2 and T3. Cross-lagged paths representing the influence of hyperarousal symptoms on sleep problems showed that hyperarousal symptoms at T1 significantly predicted sleep problems at T2 (*γ = 0.25, p<0.01*). However, hyperarousal symptoms at T2 had no effect on sleep problems at T3 (*γ = 0.07, p>0.05*). Sleep problems have no significantly predictive effect on hyperarousal symptoms from T1 to T3 (*γ = 0.04, p>0.05*; *γ = 0.01, p>0.05*).

Considering the content overlap between sleep problems and hyperarousal symptoms (having trouble falling or staying asleep), we redid a structural model analysis after omitting one hyperarousal symptom item that was conceptually related to sleep problems. The model fit was virtually unaltered (χ^2^/df = 2.998, NFI = 0.971, CFI = 0.980, TLI = 0.955, RMSEA = 0.076). The stability coefficients were 0.63, 0.12 and 0.20 for hyperarousal symptoms and 0.36, 0.12 and 0.28 for sleep problems. The cross-lagged coefficients were 0.18 and 0.07 for the effect of hyperarousal symptoms on sleep problems, and 0.06 and 0.01 for the effect of sleep problems on hyperarousal symptoms.

Overall, the findings of this study were as follows: There was a bidirectional relationship between intrusive symptom clusters of PTSD and sleep problems from T1 to T2, whereas this relation was non-significant from T2 to T3. From T1 to T3, the avoidance symptom clusters of PTSD predicted sleep problems, but were not predicted significantly by sleep problems. The hyperarousal symptom clusters of PTSD had effect on sleep problems from T1 to T2, and the effect did not exist from T2 to T3, while sleep problems have no significantly predictive effect on hyperarousal symptoms from T1 to T3. Moreover, the predictive utility between all the three symptom clusters of PTSD and sleep problems weakened with time change.

## Discussion

The present analyses were based on the data collected in a longitudinal research project that investigated the relationship between the three PTSD symptoms clusters and sleep problems over time. The findings have showed that there is a bidirectional relationship between intrusive symptoms and sleep problems from 1 year to 1.5 years, but not from 1.5 years to 2 years after the earthquake even after controlling the other two symptom clusters of PTSD and the overlapped content between intrusive symptoms and sleep problems. This extends previous findings [31], and suggests that there is a bidirectional relationship between intrusive symptoms and sleep problems in a short-time frame. Furthermore, the findings also indicated that intrusive symptoms and sleep problems could not predict each other from 1.5 year to 2 years. This is inconsistent with the emotional processing theory of traumatic stress exposure [13], [32]. Here, in a long-time frame, traumatic survivors may have got accustomed to the traumatic clues that can intrude into their cognitive world [33], and in turn decrease their degree of fears that may elicit sleep problems. Thus, the intrusive symptom clusters of PTSD didn't significantly predict sleep problems. Moreover, the findings partially support Rothbaum and Mellman' theoretical model [20], and suggest that their model can be applicable to explain the effect of sleep problems on intrusive symptoms in a short-time frame, but not in a long-time frame. It is possible that sleep problems can generate a context in which trauma survivors are more sensitive and reactive to trauma reminders [20], and short exposures to the context may further sensitize trauma reminders. However, repeated exposures to the context allow for the habituation to be enacted, which describes individuals' decreased responding to the same stimulus when it is presented repeatedly over time [34].

After controlling the other two symptom clusters of PTSD, avoidance symptoms predicted sleep problems from 1 year to 2 years after the earthquake. This is inconsistent with the previous study on the relation of avoidance symptom clusters of PTSD to sleep problems [3], but is consistent with Harvey and Bryant's (1998;1999) studies [35], [36] and supports the previous assumption that avoidance symptom clusters could predict sleep problems. Here, avoidance symptoms are the result of avoidance strategies, they always present in adolescents with PTSD [37], [38]. Once avoidance symptoms appear, adolescents would try to avoid negative emotions throughout the night [17], and this in turn interferes with their sleep onset and results in sleep problems. In addition, this result also suggests that sleep problems have no effect on avoidance symptoms at the third time wave. These results extend previous studies which suggested that sleep problems could predict PTSD [21], and demonstrate that sleep problems can only predict intrusive symptom clusters of PTSD but not avoidance symptoms and hyperarousal symptoms in a short-time frame after disaster. Taken together, these findings indicate that avoidance symptoms are predictive factors of sleep problems regardless of the time length.

After controlling the other two symptom clusters of PTSD and the overlapped content between hyperarousal symptoms and sleep problems, this study has found that hyperarousal symptoms have a significant effect on sleep problems from 1 year to 1.5 years but not from 1.5 years to 2 years after the earthquake. This is partially consistent with hyperarousal-based theories [14], [39], and suggest that hyperarousal symptoms can increase individuals' level of awareness, which may result in their sensitive responses to the external environment [40], and in turn enhance their psychosomatic awakened state, increase feelings of fear, and disturb the comfortable environment needed by adolescents' sleep. It is worth noting, however, that the assumptions of hyperarousal-based theories have not been tested from 1.5 years to 2 years after the earthquake. As increased sensitivity and sensitization of the traumatic reminders may leave the individual in a hyperarousal context that worsens over time [41], Southwick et al. have hypothesized that to calm these symptoms of hyperarousal, PTSD patients may elevate their awakening thresholds to withdraw to be awakened during sleep, which has been named the “sleep deepening” mechanism [42]. This mechanism can be dominant in a longer time frame after trauma, and may result in increased percentage of slow-wave sleep [41] but not significant sleep problems, such as difficulty in falling asleep, sleepless nights and early-morning wakefulness.

Moreover, the results found that sleep problems didn't predict hyperarousal symptoms from 1 year to 2 years after the earthquake. This is not in accord with the study result from Chang et al. [22], but parallels with that from Babson et al. [21]. Among people with PTSD, sleep problems may not increase their hyperarousal symptoms because of the already elevated levels of arousal within this population; therefore, variation in sleep quality does not impact anxious reactivity [21]. Replication and extension of the current findings will be important in increasing confidence in this inference.

This study also indicated that the relationship between PTSD and sleep problems would become weaker with time change. PTSD is associated with a failure to neutralize frightening memories [43]. This failure allows frightening memories to push in sleep hours and disturb sleep in a short-time frame. As PTSD endures for a long time, however, conditioning to environmental stimuli may decrease the relationship between PTSD and sleep problems [44], and make traumatic survivors be accustomed to frightening memories during this time, so PTSD will gradually have fewer and fewer effects on sleep problems, and even don't affect sleep problems significantly any more. In addition, the increase or maintenance of PTSD, which is a series of chronic symptoms, can be attributed to many factors under a long-time frame [45], such as cognitive rumination, social support and so on. Therefore, the relationship between sleep problems and PTSD may decrease because of many other factors' involvement in this relationship under a long-time frame. All these results have indicated that there is a close relationship between PTSD and sleep problems shortly after a traumatic event [46]. However, affected by a number of secondary factors, the relationship between PTSD and sleep problems will weaken in a long-time frame [47].

Although most of the study's theoretically derived predictions were supported, several major design and measurement limitations must be acknowledged. First, we did not investigate the adolescents' PTSD and sleep problems before the earthquake and are unable to tell the relation of PTSD/sleep problems pre-trauma to subsequent sleep problems/PTSD post-trauma. Second, we chose three items from the child behavior problems questionnaire, which may make it difficult to explore the relationship between sleep efficiency or latency and PTSD. Third, we didn't consider additional factors that may be accounted for the relations observed between adolescent's sleep problems and PTSD symptom clusters over the course of the follow-up, such as social support, cognitive rumination, secondary stressors, depression symptoms, and behavioral or pharmacotherapy for PTSD or sleep problems, and other developmental factors in adolescence.

Even with these limitations, to our knowledge, this study is among the first to examine the relationships between specific symptom clusters of PTSD and sleep problems in a longitudinal study design. Furthermore, it contributes new knowledge to previous theoretical and empirical studies on the relation of PTSD to sleep problems. From the intervention and health-enhancement perspective, the present study also provides useful information for alleviating adolescents' PTSD and sleep problems after earthquake. Regarding the findings that avoidance symptom clusters of PTSD may be important predictive factors for the development and maintenance of sleep problems in a long-time frame after the disaster, adolescents at high risk for avoidance symptom clusters of PTSD also could be at high risk for sleep problems. Hence, to improve adolescents' sleep quality under a long-time frame, school psychologists should try alleviating their avoidance symptom clusters of PTSD. Moreover, this study has also found that sleep problems have bidirectional relation to intrusive symptom clusters of PTSD within 1.5 years after the earthquake, which suggests that in a short-time frame, school psychologists can relieve adolescents' symptom of PTSD by remitting their sleep problems and vice versa.
